# The rare complication of vascular malformations of the limb after sclerotherapy: a report of 3 cases and brief literature review

**DOI:** 10.1186/s12887-023-04018-w

**Published:** 2023-04-28

**Authors:** Nianzhe Sun, Rui Liu, Gechang Cheng, Panfeng Wu, Fang Yu, Liming Qing, Lei Zeng, Xiaoyang Pang, Ding Pan, Yongbin Xiao, Umar Zeb Khan, Juyu Tang

**Affiliations:** 1 Department of Orthopedics Surgery, Hand & Microsurgery, Xiangya Hospital, Central South University, 87 Xiangya Road, Changsha, Hunan 410008 China; 2 National Clinical Research Center of Geriatric Disorders, Xiangya Hospital, Central South University, 87 Xiangya Road, Changsha, Hunan 410008 China

**Keywords:** Congenital vascular malformations, Sclerotherapy, Limb necrosis, Amputation

## Abstract

**Background:**

Vascular malformations are common but complicated types of disease in infants, with unclear causes and lack of effective prevention. The symptoms usually do not disappear and tend to progress without medical intervention. It is extremely necessary to choose correct treatment options for different types of vascular malformations. A large number of studies have confirmed that sclerotherapy has a tendency to become the first-line treatment in near future, but it is also associated with mild or severe complications. Furthermore, to our knowledge, the serious adverse event of progressive limb necrosis has not been systematically analyzed and reported in the literature.

**Case presentation:**

Three cases (two females and one male) were presented who were all diagnosed as vascular malformations and were treated by several sessions of interventional sclerotherapy. Their previous medical records showed the use of several sclerosants in different sessions including Polidocanol and Bleomycin. The sign of limb necrosis did not occur during the first sclerotherapy, but after the second and third sessions. Furthermore, the short-term symptomatic treatment could improve the necrosis syndrome, but could not change the outcome of amputation.

**Conclusion:**

Sclerotherapy undoubtedly tends to be the first-line treatment in near future, but the adverse reactions still remain major challenges. Awareness of progressive limb necrosis after sclerotherapy and timely management by experts in centers of experience of this complication can avoid amputation.

## Background

Vascular malformations are complex types of disease, which are attributed to the proliferation and malformation of blood vessels during embryogenesis with lesions of lymphatic, venous, arteriovenous, capillary, or combined origin. The common lesions are found in the head, neck, trunk, and extremities [1]. The symptoms will not disappear without medical intervention, but tend to progress over time [2]. It is a complicated and intractable disease that brings huge challenges to the disease management. However, a series of studies presented multiple treatment options about vascular malformations, including conservative management, sclerotherapy, surgery, laser therapy, cryoablation, and potential targeted therapy [3–6]. It is noted that sclerotherapy tends to be the first-line treatment of this disease, which is less-invasive, effective, and safe [6, 7]. Therefore, it is extremely necessary to understand its complications. This paper aims to present the rare and serious complication of limb necrosis after sclerotherapy in infants which is rarely systematically analyzed and published in the literature.

## Case presentation

### CASE 1

**Fig. 1:**
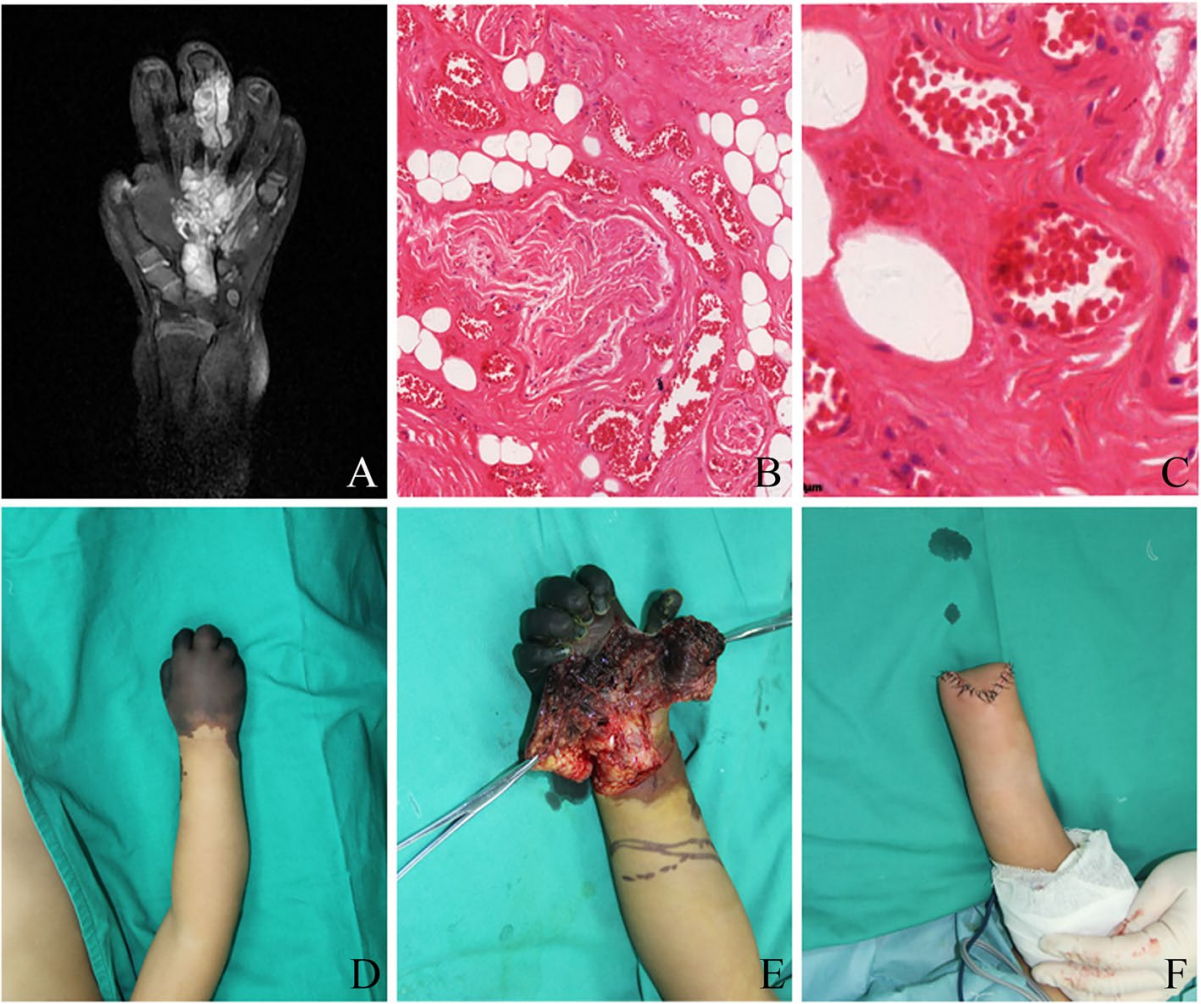
Diagnosis of primary disease and treatment after necrosis for case 1. **A**, Coronal view of T2-weighted MRI with fat-suppression sequence revealed partial hyperintensity of the right hand. **B**-**C**, Representative hematoxylin and eosin staining revealed the cirsoidangioma (B:original magnification X10, C:original magnification X40). **D**, The general picture presented the area of necrosis in wrist joint level. **E**, Intraoperative anatomy showed the deep necrotic muscle and soft tissue. **F**, The amputation photograph showed the final outcome

A twenty-eight-month-old male infant presented to our department in January 2013 for further treatment of right-hand necrosis after sclerotherapy. His previous medical records demonstrated that he was diagnosed with arteriovenous malformations existing in the second knuckle of the middle finger, the palm, and the back of the right hand for more than two years (Fig. 1A-C). Subsequently, under the guidance of ultrasound, he was treated with subcutaneous embolization using 2 ml of Polidocanol injected subcutaneously around the tumor two sessions within two weeks. Shortly after the second session, the skin temperature near the lesion decreased and the distal blood supply condition deteriorated. Symptomatic drug treatment and physical therapy temporarily controlled the progressive peripheral blood circulation disorder. Unfortunately, progressive darkening of limbs at night and family negligence prevented the child from receiving timely medical treatment. Swelling, pain, reduced skin temperature and vascular Computed tomography angiography(CTA) of the affected limb revealed embolization of the ulnar artery, radial artery and middle and lower brachial artery of the affected limb. After a period of symptomatic treatment of hyperbaric oxygen, anticoagulation, and vasodilation, the level of necrosis was basically clear at the proximal 3 cm of the wrist joint (Fig. 1D). After being transferred to our department for further treatment, medical team proposed that the condition for saving limb was not favorable. During the amputation, we found that the blood flow to the distal limb was completely cut off. The survival of limb was not possible because of extensive embolus and muscle necrosis (Fig. 1E-F).

### CASE 2

**Fig. 2:**
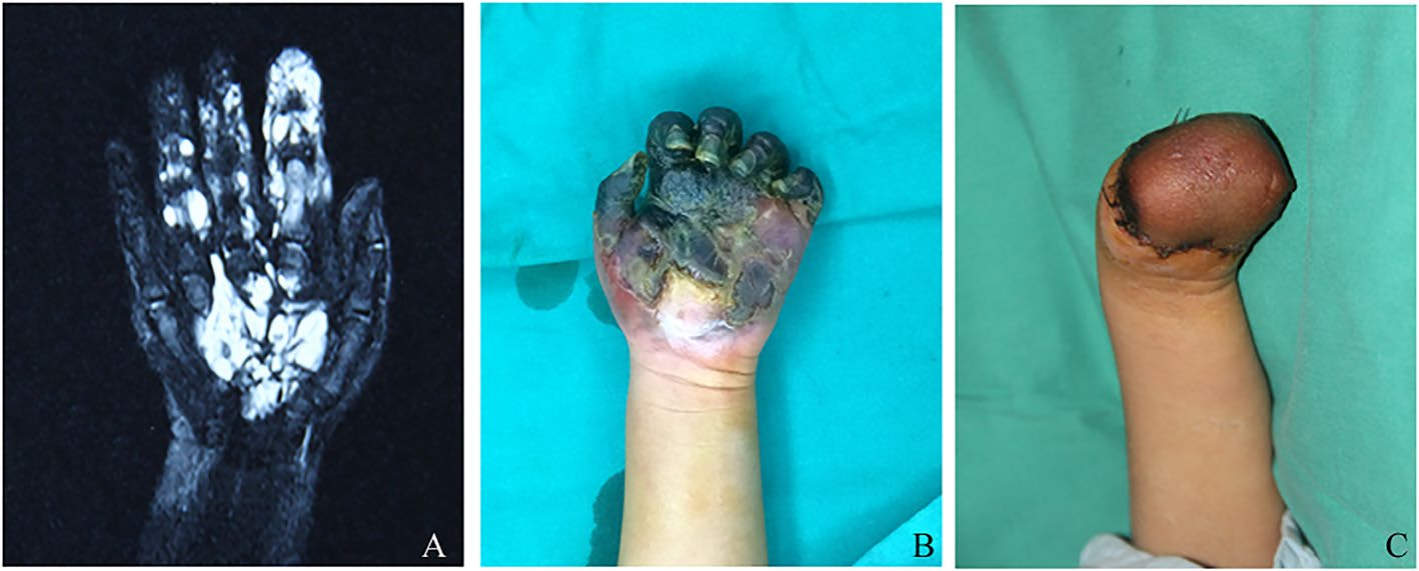
Diagnosis of primary disease and treatment after necrosis for case 2. **A**, Coronal view of T2-weighted MRI with fat-suppression sequence revealed partial hyperintensity of the left hand. **B**, The general picture presented the area of necrosis in wrist joint level. **C**, The amputation photograph showed the final outcome

A three-year-old female infant presented to our department in November 2014 for necrotic left-hand amputation. Due to a congenital purple-red plaque on the left palm for two years, she was diagnosed as having a venous malformation with Magnetic Resonance Imaging (MRI) in another hospital (Fig. 2A) and treated with sclerotherapy using 2 ml of Polidocanol injected subcutaneously around the tumor under ultrasound guidance three times. After injecting the sclerosants, tension blisters appeared at the lesion site, but the symptoms of blisters were controlled after local symptomatic treatment. Unfortunately, she was re-admitted because of the skin ulcers, increased limb stiffness, reduced skin temperature, and expanding darkening of the fingertips (Fig. 2B). The Doppler in the other hospital revealed the unclear radial and ulnar artery, weakened deep venous return, and weak arterial pulsation of the left upper limb. During the operation, we found that deep muscles and tissues had been necrotic, and the pathological sample was diagnosed as necrotic fibro-fatty tissue with hemorrhage. We eventually had to amputate the necrotic limb. The follow-up months after the operation showed that the edges of the skin on the stump were slightly darkened, suggesting that the blood flow in the distalpart of the blood vessel was not smooth, but the condition improved after conservative treatments (Fig. 2C).

### CASE 3

**Fig. 3:**
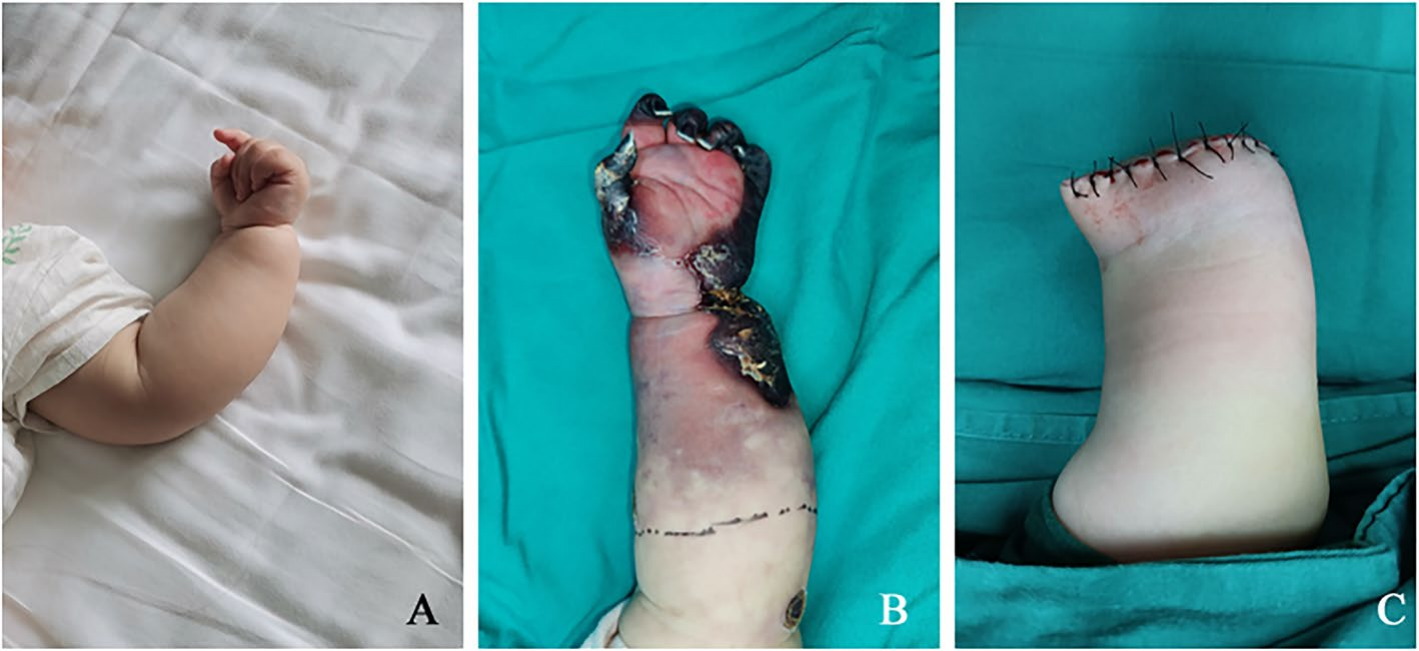
The sequential record of the affected limb for case 3. **A**, The photograph showed a more swollen left forearm. **B**, The preoperative picture presented the area of necrosis in 3 cm level at the distal end of the elbow joint. **C**, The amputation photograph showed the final outcome

**Fig. 4:**
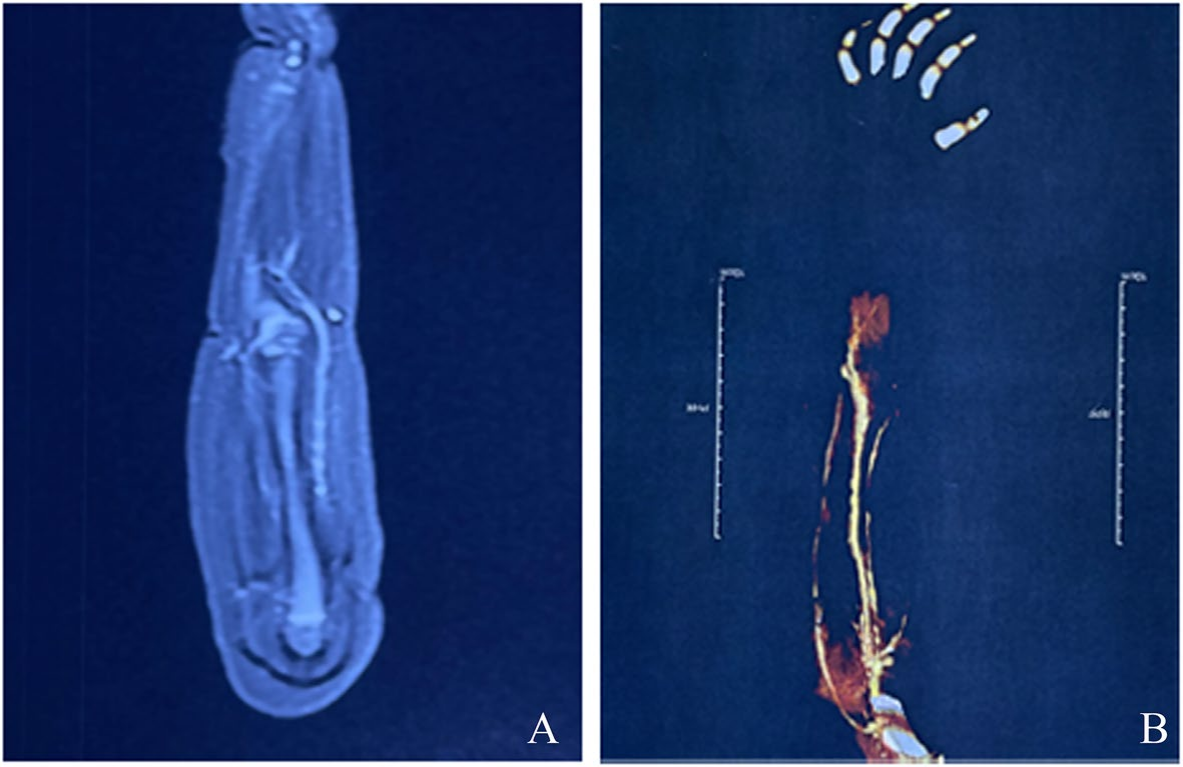
Imaging diagnosis of primary disease and necrosis. **A**, Coronal view of T2-weighted MRI with fat-suppression sequence revealed abnormally high signal points in the wrist and elbow joints of left extremity. **B**, CTA angiography showed the interrupted blood flow of the left upper limb in the middle of the left forearm

A nine-month-old female infant presented to our department in October 2021 with necrosis of left distal limb and received an amputation at middle forearm region. Four months ago, she was found with hypertrophy of left upper limb for 1 month, and then sent to a local hospital (Fig. 3A). Combined with MRI and physical examination, she was diagnosed as vascular malformations (Fig. 4A). Her previous records revealed that the second treatment with Bleomycinin in August was effective same as the first time two months ago. Under digital subtraction angiography (DSA) surveillance, arteriography of the left upper extremity was performed first, followed by arterial embolization and intraluminal injection into the deformity with scalp needle of 3 ml bleomycinmixed solution in first and second session. Imaging showed that the sclerotherapy was successful. However, limb swelling and palepalms appeared within two hours after sclerotherapy. Symptomatic treatment was taken urgently, and she was discharged from the hospital with improved condition. Unfortunately, physical examination showed skin erythema, developing into irreversible purple-black scabs, tenderness, swelling, and lowered skin temperature, which made her re-admitted to the hospital one month after surgery (Fig. 3B). CTA indicated occlusion of the ulnar and radial artery (Fig. 4B). Deep necrotic tissue was seen during the operation, and amputation was inevitable (Fig. 3C).

## Discussion and conclusions

The article presented a series of three cases who were all diagnosed as vascular malformations. All three patients (two females and one male) who were under three years old received several sessions of interventional sclerotherapy. Their previous medical records showed the use of several sclerosants in different sessions including Polidocanol and Bleomycin. The sign of limb necrosis did not occur during the first sclerotherapy, but after the second and third sessions. Furthermore, the short-term symptomatic treatment could improve the necrosis syndrome, but could not change the outcome of amputation. The details are shown in Table 1.

**Table 1:** A Brief Summary of 3 Cases

	Case 1	Case 2	Case 3
Gender	Male	Female	Female
Age to the hospital (mo)	Twenty-eight	Thirty-six	Nine
Vascular malformations type	Arteriovenous malformations	Venous malformation	Arteriovenous malformations
Lesion location	Right hand	Left hand	Left hand
Sclerosants	Polidocanol	Polidocanol	Bleomycin
Necrosis sessions	Second	Third	Second

Sclerosants are available as liquids, foam, suspensions and combined state. It can be administered into the blood stream by different routes. Generally, sclerosing agents can be classified by their physical and biological properties, which can influence treatment response and be associated with side effects [8]. The released sclerosants destroy the venous endothelium and even additional regions of the vein wall. After several sclerotherapy sessions, the vessels are transformed into a fiber cord. Parsi et al. [9, 10] published that Polidocanol could activate the intrinsic pathway of coagulation by clotting factors VIII, IX, and reduce the concentrations of blood anticoagulant factors protein S and protein C. Furthermore, the role of coagulation is associated with other parts of coagulation system, not limited to platelets and clotting factors [11]. In general, the role of transformation is played by a combine effect of sclerosing agents, including the distinct toxic damage to endothelium, degradation of blood cells, and activating blood coagulation, which all lead to pathophysiological pathway of arterial occlusion. It is worth noting that Bleomycin, an anti-tumor drug, which exploits the sclerosing action not relying on the cytostatic effect and is difficult to diffuse into the small vessel with smaller diameter, which may explain why the rare embolism usually almost appears in the large vessels [12–14].

The process by which the sclerosants work is also closely related to their adverse reactions. We summarized the following sclerosants listed in Table 2, which were commonly used for sclerotherapy of vascular malformations. Rabe et al. [15] published that the common side effects with Polidocanol are injection site hematoma, irritation, skin discoloration, and pain which were almost mild and self-limited. Consistently, the results of a high-quality randomized controlled trials (RCT) on the effectiveness and side effects of Polidocanolare similar to those listed in the Table 2 [15]. More meaningfully, a French Polidocanol study with huge person-years on long-term side effects revealed visual disturbances and muscular vein thrombosis [16]. Although Bertanha et al. [17] observerd some minor complications by a Triple-Blind RCT of Polidocanol used in the lower limbs, sclerotherapy was considered as the optional treatment of choice, provided the patient does not mind the temporary or prominent side effects. A large cohort of retrospective study conducted by Bouwman et al. [18] showed complications of using Bleomycin, Lauromacrogol, Doxycycline, Ethanol,and combinations for lymphatic malformations (LMs), and different responses and side effects ranged from different treatment combinations. An evidence based medicine study on Bleomycin for vascular malformations conducted by Horbach et al. [19] summarized the complications fever and flu-like symptoms, nausea, vomiting, and facial nerve dysfunction, which may also be related to anesthesia and intraoperative procedures. As for Bleomycin, the retrospective study published by Burrows et al. [20] and Shergill et al. [21] revealed that cellulitis, pain, swelling, skin blisters, and Horner’s syndrome were minor complications. Additionally, the side effects of other sclerosants expect candidate durgs disscussed detailedly in this article were shown in Table 2 [22–28].

**Table 2:** Summary of Adverse Events of Sclerosants

Sclerosants	Side Effects	References
Ethanol	ischemic bullae, necrosis, deep venous thrombosis, pulmonary embolism, facial nerve palsy, transient pulmonary pressure elevation, bradycardia, cardiac arrest, transmural vessel necrosis, significant edema (associated with compartment syndrome), central nervous system depression, hypertension, ulceration and pulmonary vasospasm	[15, 22–24]
STS	superficial skin blisters, hemoglobinuria, skin ulceration, skin pigmentation, local infection, neuropathy, scar, necrosis, bleeding and neovascularization	[15, 17, 18, 25–28]
Polidocanol	injection site hematoma, injection site irritation, skin discoloration, injection site pain, debilitating pain, edema, functional disability, temporary inter-digital necrosis, pigmentation, visual disturbances	[16–19]
Lauromacrogol	local swelling, hematoma, functional impairment, blistering, stridor	[16, 22]
OK-432	nerve injury, deep tissue injury, deep vein thrombosis, muscle fibrosis, airway obstruction, emergency tracheotomy, orbital decompression, infection, edema, intra-cystic hemorrhage, myalgia, and eye bulging	[23–25]
Bleomycin	Pulmonary fibrosis, scarring, hyperpigmentation	[12–14, 19, 21, 26]
Doxycycline	cellulitis, pain, swelling, skin blisters, Horner’s syndrome, hematoma, infection/abscess	[15, 20, 21, 27, 28]

*STS* Sodium tetradecyl sulfate, OK-432 (Picibanil): Suspension of Penicillin-killed *Streptococcus Pyogenes*

Due to higher incidence of necrosis and smaller compartment in limb, the use of percutaneous sclerotherapy in extremity is still controvertible. In the Birmingham experience, Mendonca et al. [29] suggested that sclerotherapy should not be considered as options of vascular malformations. Conversely, there were varieties of studies suggesting the safety and confirming successful response rate [30, 31]. Furthermore, whether there is a dose–response relationship between the incidence of adverse reactions and sclerosing agents is still controversial. Rabe et al. [32] reported that there was some evidence that the dose of sclerosants may increase the complications rate, while Guevara et al. [30] found no relation between the sclerosants volume and the incidence of complications. This is due to the occurrence of adverse reactions is not only related to the dose of the label agents, but also to its diffusion capacity, the site of injections, and the number of injections. Overall, we are still cautious about concentration and dosage. European guidelines for sclerotherapy suggested the maximum use of Polidocanol and Sodium Tetradecyl Sulphate (STS), and showed what would appear with excessive doses and high concentrations [33]. A high-quality RCT with long follow-up may investigate minor or major complications, high risk of potential syndrome, and the most optional treatment for different vascular malformations.

Every treatment is not perfect, even if research proves that the combined use of sclerosing agent is safer and more effective [34]. Bianchini et al. [7] reported that a minor adverse reaction of a transitory paresis of the posterior interosseous nerve remained, even using combinated sclerosants. Promisingly, the emergence of new state and novel compound of sclerosing agent is bringing new prospects for sclerotherapy [8, 35–37]. In addition, much deeper understanding of the molecular pathogenesis of vascular malformations makes targeted therapy response clearer by specific signaling pathways. Calver et al. [5] reported that several trials had shown efficacy of Sirolimus, Thalidomide and Bevacizumab (Avastin) in complex low-flow vascular malformations and hepatic arteriovenous malformations as immunosuppressive agents.

There are a large number of retrospective and prospective studies on adverse reactions of sclerosing agents in recent years, which indicated the lack of best optimal treatment for vascular malformations due to the imbalance between safety and effectiveness. Recent studies suggest the multiple approaches, not only the available options, but novel technology as well. Image, DSA and ultrasound guided percutaneous injection sclerotherapy have evolved as minimally invasive and effective treatments. Subhash et al. [38] revealed that guided sclerotherapy could be a smaller invasion, more safety, more effective and lower cost option in treating venous malformations. Song et al. [39] reported that there were no serious complications with DSA guided percutaneous sclerotherapy for venous malformations and this was worthy of clinical promotion.

To this end, as American Food and Drug Administration approval, we had better understand the absolute contraindications of the drug. It was not mentioned that such serious adverse reactions would kill the application prospects of such drugs. Meanwhile, further basic animal experiments yielding potential pharmacological mechanisms are necessary. Despite of rare but irreparable complications of sclerosants, we may understand advantages of sclerotherapy compared to other approaches and integrate the useful imaging technology and novel compound to avoid catastrophic adverse reactions as much as possible.

Progressive limb necrosis of vascular malformations after sclerotherapy is a rare but serious complication. It is undoubted that sclerotherapy tends to be the first-line treatment in near future, but the adverse reactions still remain major challenges. Awareness of progressive limb necrosis after sclerotherapy and timely management by experts in centers of experience of this complication can avoid amputation.

## Data Availability

All data are originated from the outpatient and inpatient medical system of Department of Orthopedics Surgery, Hand & Microsurgery, Xiangya Hospital, Central South University. The datasets used during the current study are available from the corresponding author on reasonable request.

## Abbreviations

CTA: Computed tomography angiography
DSA: Digital subtraction angiography
MRI: Magnetic resonance imaging
RCT: Randomized controlled trials
LMs: Lymphatic malformations
STS: Sodium tetradecyl sulfate
